# Microbial communities in the reef water at Kham Island, lower Gulf of Thailand

**DOI:** 10.7717/peerj.3625

**Published:** 2017-08-14

**Authors:** Naraporn Somboonna, Alisa Wilantho, Somchai Monanunsap, Suchana Chavanich, Sithichoke Tangphatsornruang, Sissades Tongsima

**Affiliations:** 1 Department of Microbiology, Faculty of Science, Chulalongkorn University, Bangkok, Thailand; 2 Genome Technology Research Unit, National Center for Genetic Engineering and Biotechnology, Pathum Thani, Thailand; 3 Marine and Coastal Resources Research Center, Lower Gulf of Thailand, Department of Marine and Coastal Resources, Ministry of Natural Resources and Environment, Songkhla, Thailand; 4 Department of Marine Science, Faculty of Science, Chulalongkorn University, Bangkok, Thailand

**Keywords:** Eukaryote, Marine, Pyrosequencing, Prokaryote, Fringing reef, 16S ribosomal RNA, Microbiota

## Abstract

Coral reefs are among the most biodiverse habitats on Earth, but knowledge of their associated marinemicrobiome remains limited. To increase the understanding of the coral reef ecosystem in the lower Gulf of Thailand, this study utilized 16S and 18S rRNA gene-based pyrosequencing to identify the prokaryotic and eukaryotic microbiota present in the reef water at Kham Island, Trat province, Thailand (N6.97 E100.86). The obtained result was then compared with the published microbiota from different coral reef water and marine sites. The coral reefs at Kham Island are of the fringe type. The reefs remain preserved and abundant. The community similarity indices (i.e., Lennon similarity index, Yue & Clayton similarity index) indicated that the prokaryotic composition of Kham was closely related to that of Kra, another fringing reef site in the lower Gulf of Thailand, followed by coral reef water microbiota at GS048b (Cooks Bay, Fr. Polynesia), Palmyra (Northern Line Islands, United States) and GS108b (Coccos Keeling, Australia), respectively. Additionally, the microbial eukaryotic populations at Kham was analyzed and compared with the available database at Kra. Both eukaryotic microbiota, in summer and winter seasons, were correlated. An abundance of *Dinophysis acuminata* was noted in the summer season, in accordance with its reported cause of diarrhoeatic shellfish outbreak in the summer season elsewhere. The slightly lower biodiversity in Kham than at Kra might reflect the partly habitat difference due to coastal anthropogenic activities and minor water circulation, as Kham locates close to the mainland and is surrounded by islands (e.g., Chang and Kut islands). The global marine microbiota comparison suggested relatively similar microbial structures among coral sites irrespective of geographical location, supporting the importance of coral-associated marine microbiomes, and Spearman’s correlation analysis between community membership and factors of shore distance and seawater temperature indicated potential correlation of these factors (*p*-values < 0.05) with Kham, Kra, and some other coral and coastal sites. Together, this study provided the second marine microbial database for the coral reef of the lower Gulf of Thailand, and a comparison of the coral-associated marine microbial diversity among global ocean sites.

## Introduction

Marine microbiota differ by ocean geography, associated animals, seasons and human influences, and are found in all marine ecosystems, from the tropics to the Antarctic and Arctic, to deep hydrothermal vents (Kari, 2007). Gilbert et al. (2012) and Somboonna et al. (2014) reported the dynamics of marine microbiomes between seasons, and day-to-night. Additionally, for a relatively identical habitats, differences in marine prokaryotic and eukaryotic populations at a small spatial scale can be due to human influences, such as from piers and residential housing (Hidayat et al., 2012; Somboonna et al., 2012).

Coral reefs have one of the greatest diversities of organisms and microorganisms on Earth. Although they cover less than 1% of the Earth’s surface, they host approximately 25% of all marine species (Bryant et al., 1998; Spalding, Green & Ravilious, 2001), and intricate and complex relationships among corals and the surrounding microorganisms and animals occur. For example, the microbes help break down nutrients, produce metabolites that protect the corals from climate change, and secrete antibiotics that can protect the corals from some pathogens (Rohwer et al., 2002; Reshef et al., 2006; Dinsdale et al., 2008; Graham & Nash, 2013). The microbial community could, therefore, impact coral health and ability to adapt to differing environmental conditions (Rohwer et al., 2002; Wegley et al., 2007; Barott et al., 2012).

As corals have been increasingly impacted by climate change and coastal human activities, they have become endangered or damaged worldwide (Brachert et al., 2013; Graham & Nash, 2013; Lins-de Barros et al., 2013). For instance, coral bleaching in areas of the Great Barrier Reef (Australia) was accompanied changes in microbial assemblages (Bourne et al., 2008). Healthy and disease coral water microbiomes have recently been extensively studied, with the aims to understand the coral associated microbial community and metabolic networks, finding a novel strategy to promote coral health via microbiota. Subsequently, this study utilized metagenomic derived 16S and 18S rRNA gene pyrosequencing to uncover the healthy fringing reef marine microbiota in the lower Gulf of Thailand, at Kham Island in Trat province, and also compared these with other global ocean sites. The study methods were established elsewhere, including by Global Ocean Sampling (GOS) Expedition and Human Microbiome Project (HMP), for examples (Rusch et al., 2007; Zinger et al., 2011; HMP, 2012).

**Figure 1:**
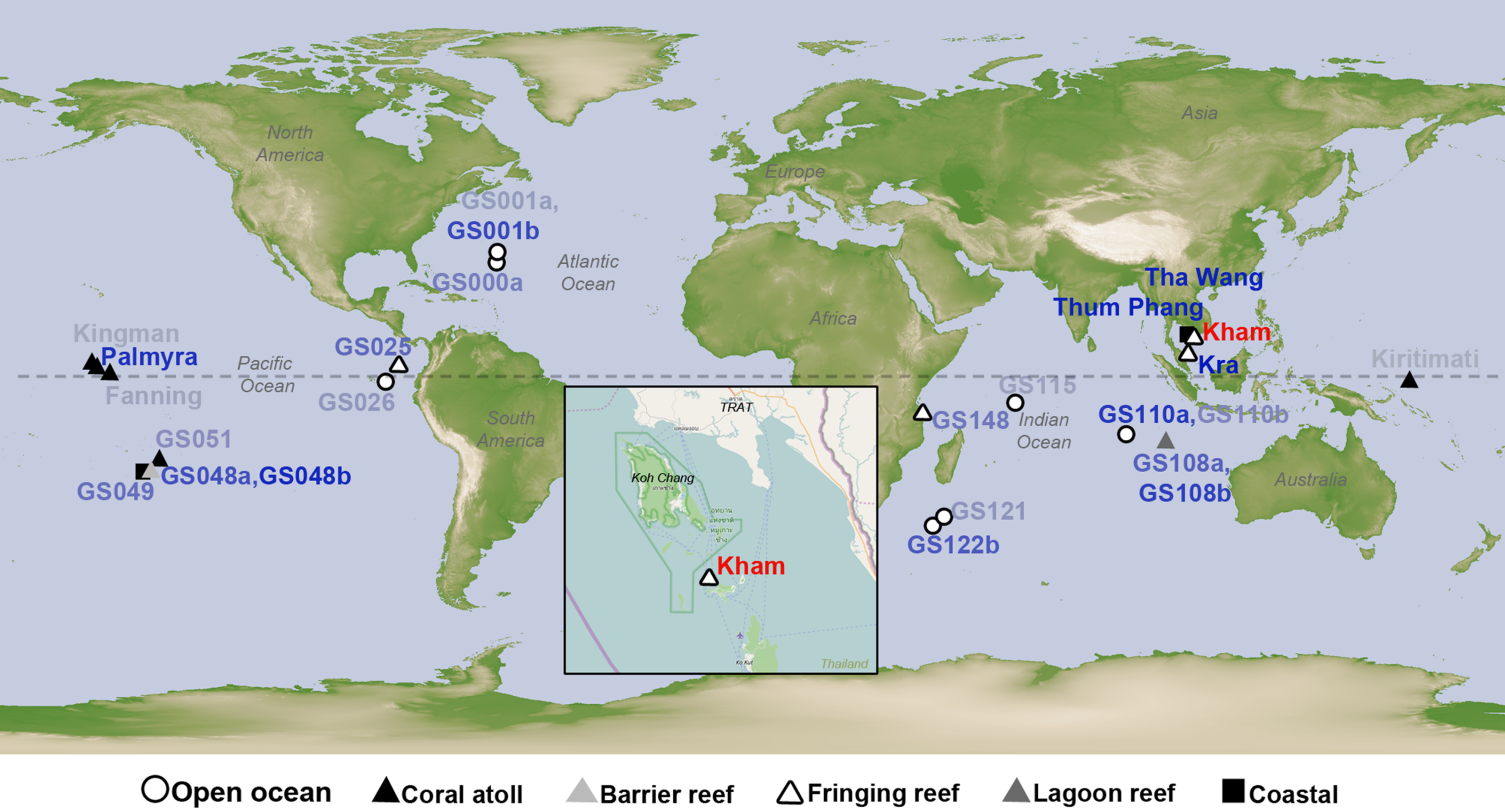
Satellite map showing the location of Kham island (red) relative to Kra, along with the other closely related marine microbial community sites, and all coral reef sites whose online microbial databases are available. Dotted line represents an equator line (0°latitude). The darker the blue font color, the closer the community similarity (based on Lennon and Yue & Clayton similarity indices) is to that of Kham. The map was from NASA Earth Observatory (public domain) (http://earthobservatory.nasa.gov/), with the inset Kham map from OpenStreetMap (http://www.openstreetmap.org/), retrieved on 13 December 2016.

Coral reefs worldwide are categorized into four types: fringing reef (the most common type), barrier reef, lagoon reef, and coral atoll. Thailand and regions in tropical waters have naturally occurring fringing reefs, which are characterized by a long-term reef growth process from an attached shoreline. Recently, we reported the marine microbial diversity for the fringing reef water at Kra Island in the lower Gulf of Thailand (Somboonna et al., 2014). The coral habitat and condition at Kham and Kra are parallel (Lins-de Barros et al., 2013), except Kham locates 14 km closer to the shore (Fig. 1), which might have some impact on the microbial diversity due to human activities (e.g., snorkelling and fishing) and minor water circulation (i.e., surrounded by mainland and islands) (Lins-de Barros et al., 2013). The coral reef size at Kham was ten-fold smaller than at Kra (Kham ∼56,000 m^2^, Kra ∼560,000 m^2^).

## Materials & Methods

### Sample collections

Water samples were collected into separate sterile containers from 1 m above the coral reefs, equal to approximately 1 m below the seawater surface, at Kham Island on two occasions, once in May 2011 and once in January 2012, between 11:00–14:00 h. This represents the summer and winter, or dry (low tide) and wet (high tide) seasons, respectively. Of note is that the sample collection methods (including sampling depth), as well as the sampling period for summer and winter seasons, and the sampling time of the day, between Kham and Kra, were identical (Somboonna et al., 2014). A minimum of three independent seawater samples of 5 L each was collected per time. The GPS coordinates of these independent sampling sites were N6.97224 E100.85620, N6.97383 E100.85691, N6.97443 E100.85875 and N6.97321 E100.85862. These sites were approved and the samples were collected with the support by the Marine and Coastal Resources Research Center, Lower Gulf of Thailand (MCRRC). This research area does not require ethical approval. Information of general water properties, including the pH, salinity, temperature, dissolved oxygen (DO) and total suspended solids (TSS), at Kham on the sample collection data and time, were provided by the MCRRC using YSI650 Multiparameter Display System (YSI Inc., Yellow Springs, OH, USA) following manufacturer’s protocols (Somboonna et al., 2014). All samples were transported on ice and processed immediately upon arrival in the laboratory.

### Metagenomic extraction and quality examination

Each water sample was poured through a four-layered sterile cheesecloth to remove particles of >30 µm (Somboonna et al., 2012). Then, free-living microorganisms and particles of ≥0.22 µm in size were captured using a sterile 0.22-µm filter (Merck Millipore, Billerica, MA, USA). Total nucleic acids were extracted, and appeared at around 40 kb in size, using the Metagenomic DNA Isolation Kit for Water (Epicentre, Madison, WI, USA) (Begum & Murray, 2008). The extracted metagenomes were analyzed for quality and concentration by agarose gel electrophoresis and A_260_/A_280_ nanodrop spectrophotometry.

### Preparation and pyrosequencing of the pre-tagged 16S and 18S rDNA fragment libraries

For broad-range amplification of the prokaryotic 16S and eukaryotic 18S rRNA genes, the universal 338F and 803R primers, and 1A and 516R primers were used (Somboonna et al., 2014). An eight-nucleotide barcode was added to both ends of each primer pair to label each sample, following the established protocols (Table S1) (Meyer, Stenzel & Hofreiter, 2008). These primer sequences are accepted worldwide to generate the 16S and 18S rDNA libraries for pyrosequencing (Somboonna et al., 2012; Somboonna et al., 2014; Mhuantong et al., 2015). The PCR recipe for the respective 16S and 18S rDNA library construction comprised 1 × EmeraldAmp^®^ GT PCR Master Mix (TaKaRa, Shiga, Japan), 0.3 µM of each barcode-appended primer, and the metagenomic template. The thermocycling conditions were 95 °C for 4 min, and 30 cycles of 94 °C for 45 s, 50 °C for 55 s and 72 °C for 1 min 30 s, followed by 72 °C for 10 min (Somboonna et al., 2014). The products of approximately 466 (16S rRNA) and 560 (18S rRNA) nucleotides in length were agarose gel purified using PureLink^®^ Quick Gel Extraction Kit (Invitrogen, Carlsbad, CA, USA). Negative control of each library construction step included no template control (sterile water in replace of metagenomic template), and no PCR amplicon was found. The independent replicate samples were pooled, ligated to 454-sequencing adaptors, and pyrosequenced on an eight-lane Roche picotiter plate using the 454 GS FLX system (Roche, Branford, CT, USA) at the in-house facility of the National Center for Genetic Engineering and Biotechnology (Pathum Thani, Thailand). Pyrosequencing was performed following the manufacturer’s protocols.

### Sequence quality screening and annotation

Sequences that did not pass the default threshold quality of the pyrosequencing screening software (e.g., ambiguous base, homopolymer >8 bp, chimera sequence), or were shorter than 50 nucleotides excluding primer and barcode sequences, were removed. The rest were categorized by sample names based on the eight-nucleotide barcode sequences. Species were annotated by BLASTN (Altschul et al., 1997) with a ≤10^−5^  *E*-value cut-off, using the NCBI non-redundant (Sayers et al., 2010), RDP (Maidak et al., 2001) and Greengenes (McDonald et al., 2011) databases for prokaryotes, and the NCBI non-redundant (Sayers et al., 2010), EMBL (Leinonen et al., 2011) and SILVA (Pruesse et al., 2007) databases for eukaryotes. The number of passing quality and annotated sequences of Kham 16S rDNA (summer) and 18S rDNA (summer and winter) each are all above 3,000 sequences. Operational taxonomic units (OTUs) were defined at a phylogenetic distance (0.20 for phylum, 0.15 order, 0.10 family, 0.05 genus, and 0.03 species) based on the 16S rRNA V3–V4 sequences by Mothur with default parameters (Schloss & Handelsman, 2004; Schloss & Handelsman, 2005). Sequencing depth was analyzed by Good’s coverage index, which estimate a biodiversity coverage of the sample to the true biodiversity, and rarefaction analysis that estimates the number of annotated sequences to the estimate species richness of the community (Schloss & Handelsman, 2005). The rarefaction curve reaches plateau, inferring the sufficient sequencing depth.

### Community comparison analysis

Phylum (and genus) percent composition was computed as the frequency of reads in the phylum (or genus) divided by the total number of the identified reads. The percent phylum composition was displayed as bar chart using EXCEL. The genus composition was displayed as heatmap by MATLAB, using a range of colors to represent different percent abundances. The Kham’s prokaryotic community composition was compared with those of the 73 GOS ( https://imicrobe.us/project/view/26) (Rusch et al., 2007; Zinger et al., 2011), four coral sites of Northern Line Islands (Fanning, Kiritimati, Palmyra and Kingman) (Dinsdale et al., 2008), and two coastal and one coral sites of Thailand (Somboonna et al., 2012; Somboonna et al., 2014). The compared databases contained the 16S rDNA profiles representing prokaryotic microbiota that were obtained by parallel experimental methods; metagenomics derived 16S rDNA sequencing or metagenome sequencing. The 16S rRNA V3–V4 region amplified by the universal primers in this current study was widely accepted (Somboonna et al., 2012; Somboonna et al., 2014; Klindworth et al., 2013; Takahashi et al., 2014). The coastal and coral sites of Thailand also contain the 18S rDNA profiles allowing the eukaryotic microbiota comparison. The global ocean 16S rDNA profiles comparison allowed unweighted pair group method with arithmetic mean (UPGMA) clustering of prokaryotic community patterns by Yue & Clayton diversity indices and the average pairwise community similarity matrics (e.g., Morisita-Horn, Jaccard, and Yue & Clayton dissimilarity indices), at OTUs level of 0.03 (species) using Mothur and newick-formatted dendrogram with default parameters (Chao et al., 2006). These calculations served cross-validation of the community similarity computations. GC content of each microbiota was calculated from the 16S rDNA profile of each. Further, as GPS of some sites allow shore distance calculation, and seawater temperature and salinity data were available (Rusch et al., 2007; Dinsdale et al., 2008; Zinger et al., 2011; Somboonna et al., 2012; Somboonna et al., 2014), the prokaryotic communities correlation with these factors were analysed by Spearman’s correlation at 0.20 cut-off level against the principal coordinates analysis (PCoA) computed from Thetayc community similarity scores. As each bacterium or archaea harbours a set of metabolic potentials, which data of each bacterial metabolic capacities are available in mg-RAST (Meyer et al., 2008), the metabolic potential of one’s prokaryotic microbiota was derived from its community compositions (Overbeek et al., 2005; Aziz et al., 2008; Somboonna et al., 2014). The mg-RAST functions are categorized into major subsystems and functional groups (Meyer et al., 2008).

## Results

### General water qualities

The Kham’s fringing reef seawater was clear and had no abnormal smell, and the coral condition was normal at times of sample collection (data not shown). The (pH, salinity, temperature and DO) were relatively constant between the summer and winter seasons, except an amount of TSS that was greater in the winter than the summer (Table 1). This might correlate with the average concentrations of microbial metagenomes in summer and winter that were 0.16 and 0.28 ng/mL of seawater, respectively.

**Table 1:** General water properties.

Seasons	pH	Salinity (psu)	Temperature (° C)	Dissolved oxygen (mg/L)	Total suspended solids (mg/L)
Summer	6.4	31.0	30.8	6.2	22.4
Winter	6.5	32.0	29.1	5.8	30.8

### Prokaryotic compositions

OTUs were defined, and the sampling depth was analyzed by rarefaction curves and Good’s coverage index. Rarefaction curves, which estimate the number of annotated sequences to the estimate genus richness, of Kham summer reaches plateau inferring the sufficient sequencing depth, and lie in the range that was published elsewhere (Fig. S1) (Aguilar et al., 2016; Duniere et al., 2017). The Good’s coverage index that estimates the percent coverage to the true biodiversity supports the rarefaction analysis (97.45% at phylum level).

**Figure 2:**
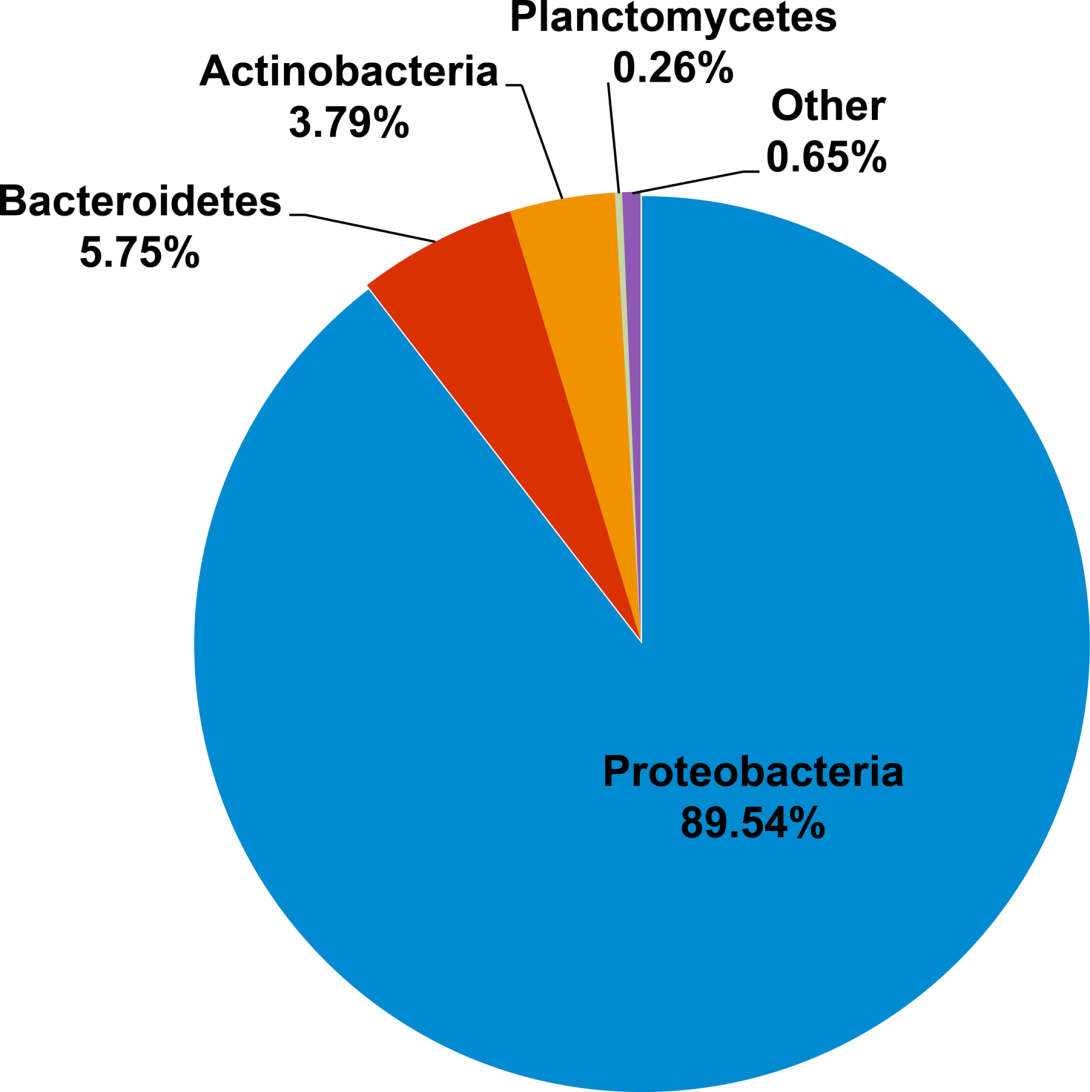
Prokaryotic phyla compositions for Kham reef surface water in the summer.

**Figure 3:**
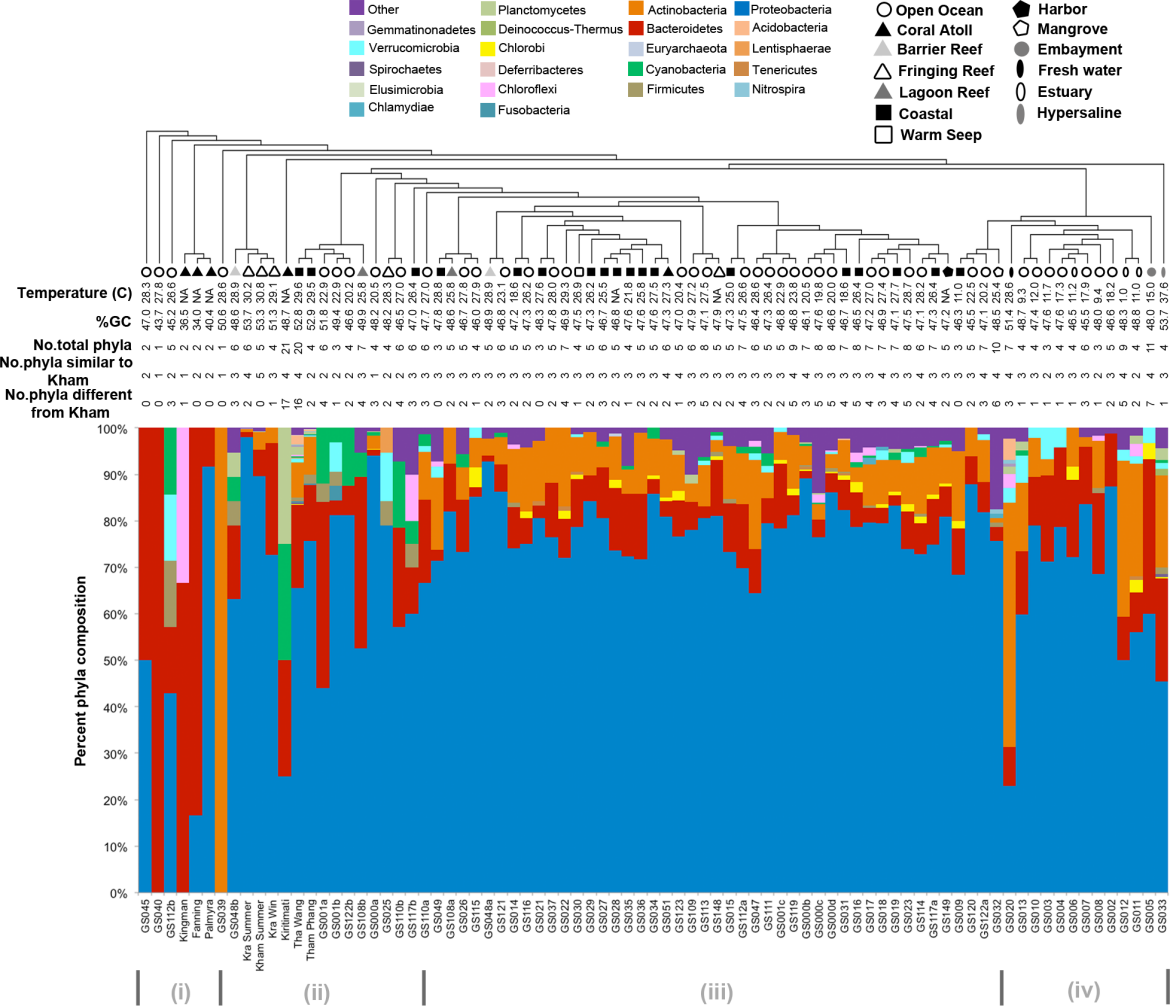
Phylum-level taxonomic compositions of prokaryotes at Kham reef surface in summer, compared with the other global ocean sites. Each community was phylogenetic clustered based on their similarity metrics. Following information include water temperature, %GC, total number of phyla for each site, and the number of phyla that are shared and different to that at Kham. Due to limited space, the tree branch does not represent evolutionary distance among communities. Different color denotes different phylum.

Kham showed Proteobacteria as the major phylum, followed by Bacteroidetes, Actinobacteria, uncultured bacteria in GN02 and Planctomycetes (Fig. 2). Five phyla diversity falls within an average phyla diversity of the global coral sites (Fig. 3: 5.77 phyla/site). The GC content of this prokaryotic community was found 53.3%, close to that for Kra in summer (53.7%), but was higher than the average GC of global ocean sites (46.46%) (Fig. 3).

**Figure 4:**
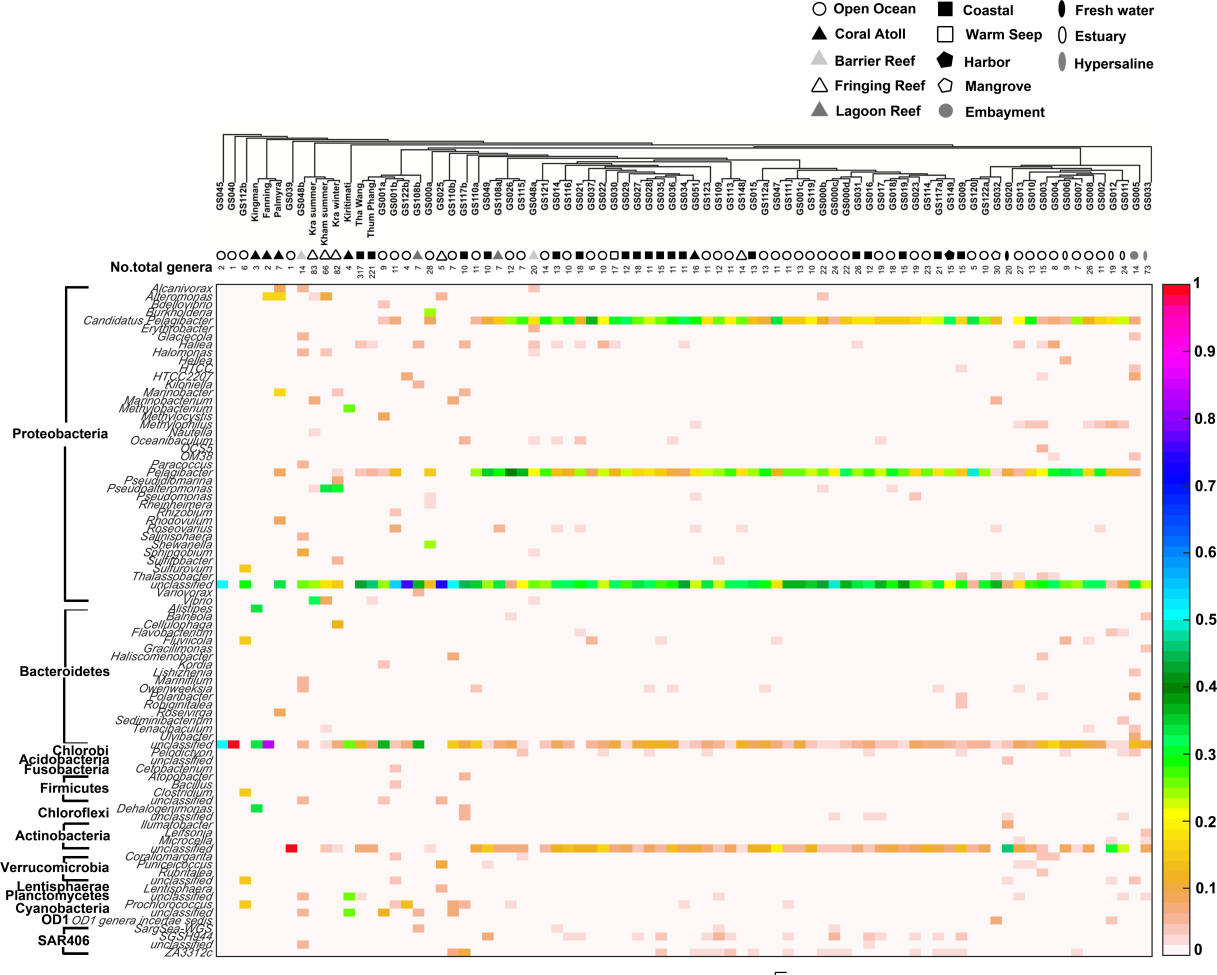
Genus-level taxonomic compositions of prokaryotes at Kham reef surface water in summer, compared with the other global ocean sites. Each community was phylogenetic clustered based on their similarity metrics, followed by the total number of genera for each site. The tree branch does not represent evolutionary distance among communities. Genus and species with less than 3% abundance were excluded in the figure, and genera of the same phylum were bracketed. Different color denotes a different percent composition of that genus, based on the color degree on the right (i.e., aqua is 0.5 or 50%).

Comparison of the prokaryotic compositions between the Kham (summer) and Kra (summer and winter) datasets exhibited several shared bacterial genera (Fig. 4). The pairwise dissimilarity metrics by Morisita-Horn and Thetayc between Kham and Kra were small, compared with the pairwise community comparisons with other global sites (total comparison is 75 global sites) (Table S2). The dissimilarity index ranges 0 to 1, in which the closer to 0 means the closer the pairwise community similarity is. Subsequently, the data indicated the community structure at Kham (summer) was closer to that at Kra in winter than at Kra in summer (Figs. 3 and 4).

## Global comparison of marine prokaryotic microbiota and the metabolic potentials

The species composition of the microbiota at Kham at the phylum and genus levels were compared with the available global datasets that include five coral atolls (Kingman, Fanning, Palmyra, Kirtimati and GS051), one barrier reef (GS048), three fringing reefs (Kra, GS025 and GS148), one lagoon reef (GS108) and 73 Global Ocean Sampling (GOS) sites. According to the community similarity statistics and as displayed in dendrograms in Figs. 3 and 4, the Kham (summer) microbiota was close to that for Kra in winter and in summer, followed by GS048b (1.4-m barrier reef water, Cooks Bay, Fr. Polynesia), Palmyra (∼11-m atoll water, Northern Line Islands, United States) and GS108b (1.8-m lagoon reef water, Coccos Keeling, Australia), respectively. Figure 1 described their geographic correlation nearby an equator line. Comparing seawater temperatures among datasets found Kham and Kra sharing the high water temperature and %GC content (Fig. 3). Note that ‘a’ and ‘b’ in GS048a and GS048b represent the same sample site and depth, but ‘a’ represents the metagenomic extraction of microorganisms of diameter sizes 0.1–0.8 µm and ‘b’ represents the metagenomic extraction of microorganisms of diameter sizes 0.8–3.0 µm (Rusch et al., 2007; Zinger et al., 2011). Bacteria are generally greater than 1 µm in diameter, so the similarity of the microbiome at Kham to those at GS048b and GS108b made sense.

Moreover, the phylum diversity across global ocean habitats in Fig. 3 could be visualized into four broad clusters based on dendrogram branching: (i) from GS045 to Palmyra, containing a high percent of Bacteroidetes (branch levels 1 to 3); (ii) approximately GS048b to GS117b, containing a mix of Bacteroidetes, at a higher percent, with some Cyanobacteria, Planctomycetes and Firmicutes (branch levels 4 to 8 split left the first four levels); (iii) approx. GS049 to GS032, containing a roughly equal proportion of Actinobacteria and Bacteroidetes, followed by unclassified other species (branch level 8 split left from levels 4 to the rest, to the left three branches of level 8 split right); and (iv) approx. GS020 to GS033, with the relatively high percent of Actinobacteria or Bacteroidetes (the rests of branch level 8 split right and branch level 7 split right). The coral reef marine microbiota lie mostly in cluster (ii) for ∼1 m reef surface water, and cluster (i) for ∼10 m coral atoll water (Fig. 3). One major differing genera between coral vs. other ocean sites were *Candidatus* Pelagibacter and *Pelagibacter* (both represent a member of the SAR11 clade) in Proteobacteria. Both genera were absent, or minor, in most coral sites (Fig. 4).

**Figure 5:**
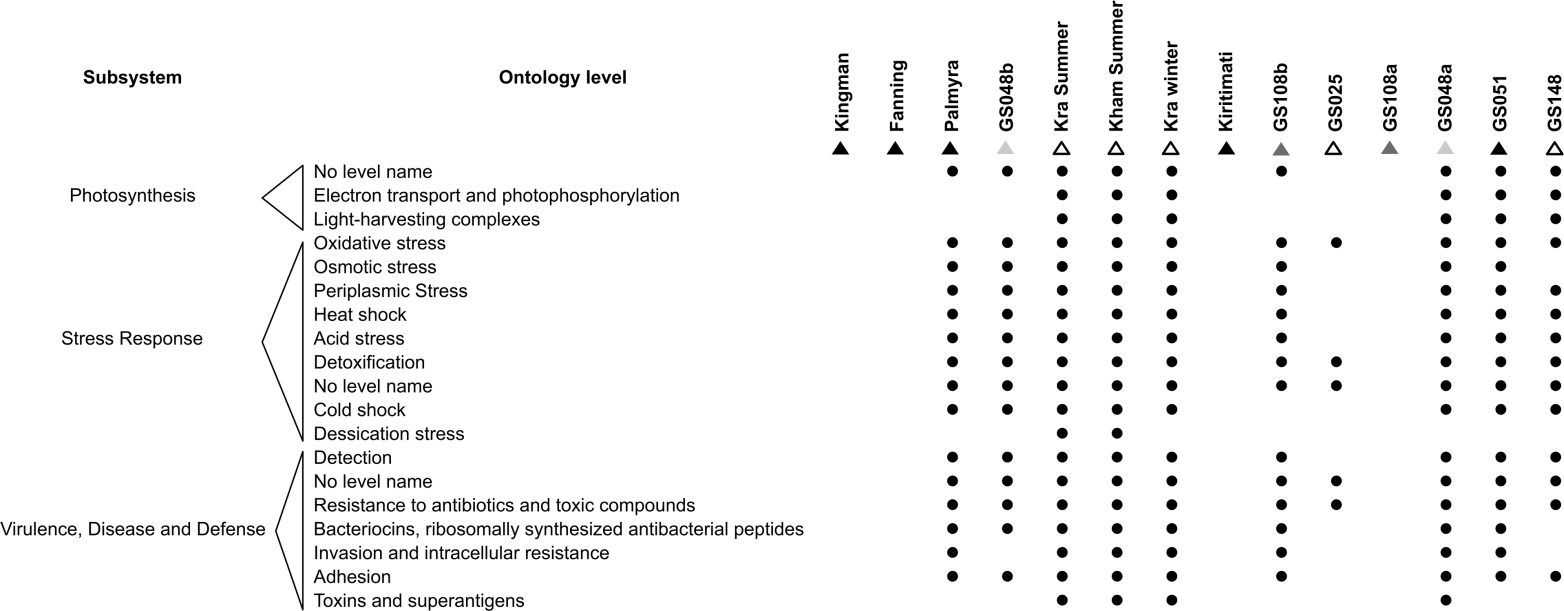
Metabolic potentials of the prokaryotic communities among coral reef sites, categorized by subsystems and functional groups (ontology level).

As microorganisms each harbour a set of metabolic capabilities, together create the microbiota’s metabolic potentials. Figure 5 describes the metabolic potentials of the different coral reef microbial communities at the major subsystems and functional ontologies. Kra, Kham, GS048a, GS051, GS108b, Palmyra and GS048b demonstrated nearly all functional ontologies. Indeed, Kham only missed 3–5 functional groups of total denoted in Kra (Fig. S2).

### Eukaryotic compositions of Kham summer and winter

**Figure 6:**
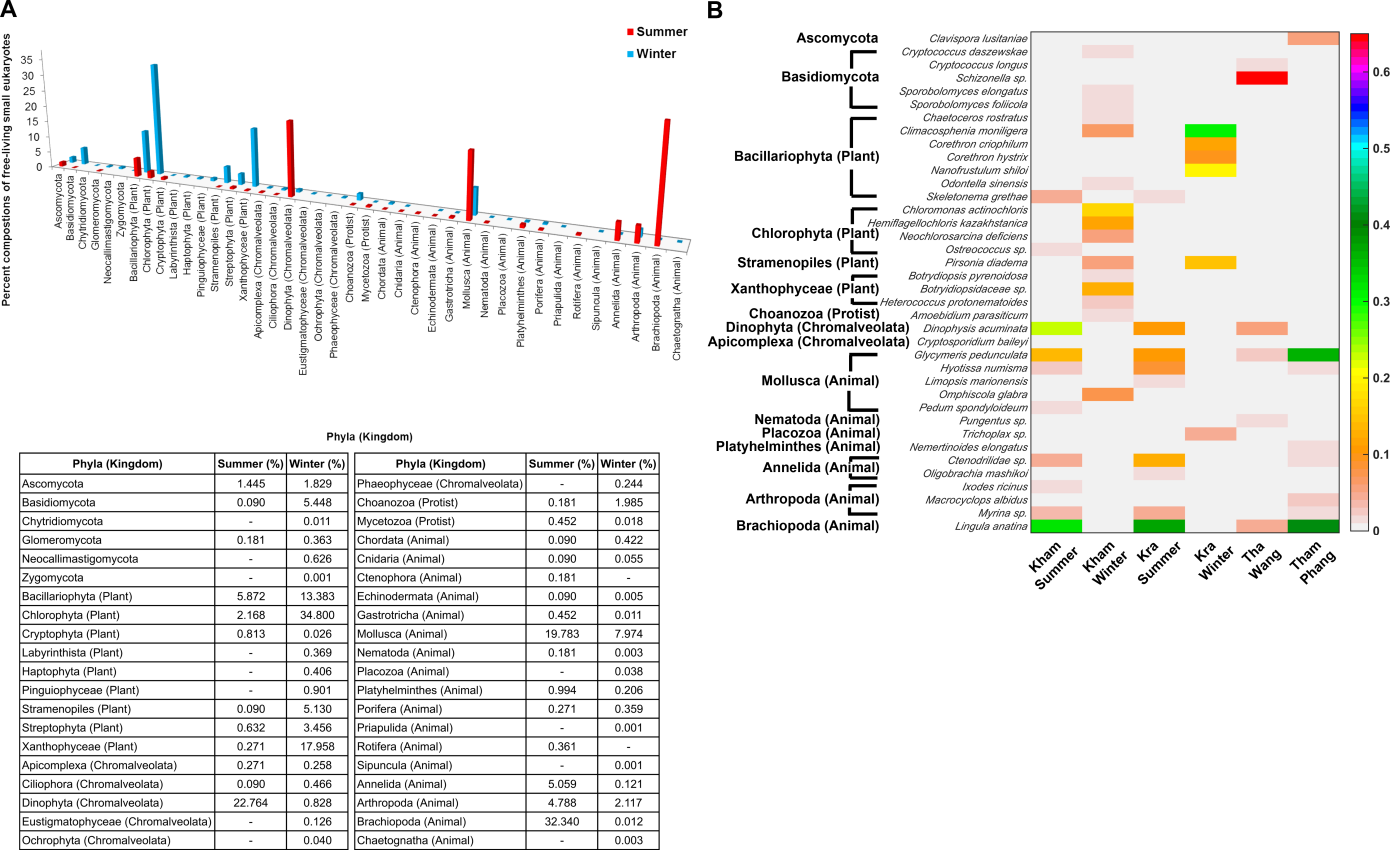
Taxonomic compositions of the microbial eukaryotes in Kham reef surface water in the summer and winter, shown at the (A) phylum and (B) genus level. Phylum other than fungi has the kingdom denoted in parenthesis. In (B), different color denotes a different percent composition of that genus, based on the color degree on the right (i.e., pink is 0.6 or 60%). Of each community, genus and species with less than 1% abundance were excluded in the figure.

The free-living marine eukaryotic species in the summer and winter seasons at Kham were analysed. The data demonstrated the microbial eukaryotic diversity between seasons. In addition to the disparate phylum evenness between the seasons, the winter was indicated the more phylum richness than the summer: an additional three, three and two phyla of fungi, plants and animals, respectively, were found in the winter (Fig. 6A). A newly classified kingdom of Chromalveolata was also found (Adl et al., 2012), and although sore phyla of this kingdom were denoted in the winter (i.e., Eustigmatophyceae, Ochrophyta and Phaeophyceae), an exceedingly high proportion of phylum Dinophyta were present in the summer (Fig. 6A). Chromalveolata have characteristic morphologies somewhere among plants, protists and fungi. For instance, chromalveolates have cell walls and photosynthetic ability like plants, yet have slime and mold characteristics like fungi (Adl et al., 2012). These new creatures capable of crossing kingdom characteristics may underlie special requirements in the summer coral habitat. The greater proportion of fungi and plant species were in the winter, whilst there were more animals in the summer (Fig. 6A). Dominated genera in the winter were *Cryptococcus*, *Sporobolomyces*, *Chaetoceros*, *Climacosonenia*, *Chloromonas*, *Hemiflagellochloris*, *Neochlorosarcina*, *Pirsonia*, *Botryidiopsidaceae* and *Heterococcus*, and many were consistent with the Kra winter profile (Fig. 6B). The similar trend between the Kham and Kra eukaryotic community structure changes in summer and in winter confirmed the seasonal dynamics of the marine eukaryotes, given the change in the community structure likely affects the species-species interaction and hence the metabolic networks in a way to intercalate with the coral ecosystems in each season, respectively (Wegley et al., 2007; Barott et al., 2012; Somboonna et al., 2014).

## Discussion

This study successfully obtained marine microbial prokaryotic and eukaryotic databases for one naturally healthy fringing reef site in the lower Gulf of Thailand (Kham Island). This helps fulfil the knowledge of coral reef marine microbiome ecology, and perhaps points to coastal effects on the microbial diversity at Kham compared to the more open-sea Kra locality (Fig. 1).

For the prokaryotic community, bacteria of phyla Proteobacteria, Bacteroidetes, Actinobacteria, Planctomycetes and uncultured bacteria in GN02 (Fig. 2) are common in Kham, as well as in the prokaryotic profiles of Kra in the summer and winter seasons (Somboonna et al., 2014), and also of global coral sites (Wegley et al., 2007; Gilbert et al., 2012). No archaea was found (Fig. 2) (Wegley et al., 2007; Gilbert et al., 2012; Somboonna et al., 2014). Because archaea were previously reported common in contaminated (e.g., metal or oil) or polluted marine sites (Biers, Sun & Howard, 2009; Somboonna et al., 2012), no finding in undisturbed coral site like Kham is suggestive of the undisturbed condition of Kham waters. In details, the phylum and genus compositions of Kham (summer) were between the summer and winter dynamics of Kra (Figs. 3 and 4). This might involve some dissimilar water properties between Kham and Kra (Somboonna et al., 2014), such as the much higher amount of TSS in Kham, and also in an opposite trend to the report in Kra where Kham has the lower level of TSS in the summer than the winter (Table 1). The relatively lower TSS in Kham in the summer might be associated with the human water and coastal activities that are generally fewer in the summer than the winter. In Thailand, the tourist season is in the winter due to the hot climate in the summer. Supportively, human-associated bacteria, such as *Staphylococcus aureus* and *Escherichia coli*, were rare reported in the summer: *S. aureus* 0% and *E. coli* <1% (Fig. 4: <3% of abundance is not shown in the figure).

To determine if factors of shore distance, seawater temperature and salinity may affect the community composition, the Spearman’s correlation analysis against the PCoA of the communities relation was additionally performed. The results indicated potential correlations between community memberships and shore distance (*p*-value 0.035), and seawater temperature (*p*-values = 0.025), in a direct direction with coral sites at Kham, Kra, GS025 and GS048b, and coastal sites Tha Wang and Tham Phang. The correlation with salinity was not found (*p*-values >  0.05). Nonetheless, because the *p*-values for shore distance and seawater temperature were still >0.01, we reported as “potential” correlation (Fig. S3: gray vectors).

Analysis of the main sources of coral-killing pathogens, such as *Serratia marcescens*, was not detected in either Kham nor Kra (Fig. 4). This white pox coral causing disease outbreak has affected shallow coral reefs throughout the Caribbean (Krediet et al., 2009). An absence of coral pathogens might involve many genera in Kham, such as *Haliea, Pseudoalteromonas* and several genera in Actinobacteria (Fig. 4), that confer antibacterial properties and thus keep the pathogens in low prevalence (Shnit-Orland, Sivan & Kushmaro, 2012; Zhang et al., 2012; Manivasagan et al., 2013).

To attempt visualization of the marine microbial pattern that might represent tropical fringing reef ecosystems, we compared the Kham, Kra, and other global coral reef and ocean sites microbiota, and found the coral marine microbiota of varying reef types and climates (latitudes) share an extent of similarity, supported by community similarity indices and UPGMA dendrogram clustering (Figs. 1 and 3). The result highlights that some microorganisms that result in the broad clusters (ii) and (i), in Fig. 3, might be essential to coral reef ecosystems. In contrast, the microbiomes of open ocean and coastal sites shared the community pattern (Fig. 3: cluster (iii)), while estuary, hypersaline and freshwater microbiomes shared the other pattern (cluster (iv)). A combination of phyla Proteobacteria, Bacteroidetes, Actinobacteria and Planctomycetes were reported on coral reef microbiomes (Fig. 1) (Webster & Bourne, 2007; Neulinger et al., 2008; Li et al., 2013). While Cyanobacteria were found in some coral reef sites (e.g., GS048b and Kiritimati), the absence or minor presence in Kham and many reef sites, including Kra, Kingman, Fanning and Palmyra (Figs. 3 and 4), does not suggest the lack of photosynthetic activity at these sites. Other photosynthetic bacteria must be present, since the metabolic potentials of Kham and Kra, the lower Gulf of Thailand, showed all photosynthetic activities (e.g., light harvesting complexes, and electron transport and photophosphorylation) (Fig. 5). On another note, no to minimal presence of a clade of uncultured SAR11 in coral reef sites, including Kham and Kra, while open ocean sites are (Meyer et al., 2009). The SAR11 was reported adapted to minimize nutrient use, to suit living in such a limited nutrient environment as open ocean (Morris et al., 2002; Dufresne, Garxzarek & Partensky, 2005; Giovannoni et al., 2005). Because the coral sites are relatively nutrient rich compared with open ocean, no to small presence of the SAR11 was. In supportively, the metabolic potentials of the coral reef microbiota in Kham and Kra were diversely fruitful (Fig. 5, Fig. S2). The diverse marine microbial metabolisms may promote the coral resistance to global warming and coral bleaching (Rohwer et al., 2002; Klaus et al., 2005; Reshef et al., 2006; Wegley et al., 2007).

In addition, the marine eukaryotic profiles in the summer and winter seasons of Kham were analyzed, and demonstrated seasonal dynamics that were correlated with those from Kra (Fig. 6A) (Somboonna et al., 2014). This seasonal eukaryotic pattern may support the monsoon influences in the Gulf of Thailand. Tang et al. (2006) reported the increased detection of photosynthetic organisms during the winter in the Gulf of Thailand was associated with the monsoon patterns. The more plant species in the winter could support nutrient-cycling and photosynthetic activities to the corals (Fig. 6B) (Barott et al., 2012; Bourne et al., 2013). The finding of Chromalveolata might pose a hazard as some of which are pathogenic phytoplankton. The marine plankton *Dinophysis acuminata* in the summer is of concern, since *D. acuminata* can secrete toxic okadaic acid that causes diarrhetic poisoning to the consumed animals. This disease outbreak has been reported among mussels and flounder species harvested from this area in the late spring to summer, consistent with our seasonal finding (Lee et al., 2011; Naustvoll, Gustad & Dahl, 2012; Diaz et al., 2013).

## Conclusions

This report compares the coral reef marine microbiota in the lower Gulf of Thailand, between Kham and Kra Islands, with 13 other coral sites and GOS sites, which unveiled some patterns of diversity that might be essential to the coral reef ecosystem. However, more coral sites and time-series based analyses, as well as the metagenomic analysis, are required to acquire the information essential to monitor and help conserve endangered corals, for instances the development of coral reef status biomarkers from the environmental abiotic parameter and specific marine microorganisms that show statistical correlation.

##  Supplemental Information

10.7717/peerj.3625/supp-1 Figure S1 Rarefaction analysis at distance level of 0.20 (A, phylum), 0.10 (B, family), 0.05 (C, genus) and 0.03 (D, species) Different color lines represent the number of annotated 16S rDNA sequencing reads in other published data (Tha Wang, Tham Phang, Kra summer, Kra winter, Northern Line Islands, and random selected GOS).

10.7717/peerj.3625/supp-2 Figure S2 Spearman’s correlation analysis of community associations with shore distance, seawater temperature and salinity Metabolic potentials of the prokaryotic communities among coral reef sites, categorized by subsystems and functional groups (ontology level).

10.7717/peerj.3625/supp-3 Figure S3 Spearman’s correlation analysis of community associations with shore distance, seawater temperature and salinity Different color dots denote different types of ocean habitats, e.g., fringing reef, barrier reef, lagoon reef, and coastal. The direction of vector infers the direction of the effect, the length infers the strength of the association in that direction, and the color grey indicates the level of 0.01 < *p*-value ≤0.05. Vector for salinity has *p*-value >0.05, and thus was omitted.

10.7717/peerj.3625/supp-4 Table S1 Pyrotagged 16S and 18S rRNA genes universal primers Italic sequence denotes 8-nt pyrotagged sequence.

10.7717/peerj.3625/supp-5 Table S2 Pairwise dissimilarity matrics of Kham summer (A) 16S rDNA and (B) 18S rDNA profiles, to the top 5 highest similarity matric sites The dissimilarity index ranges 0 to 1, in which the closer to 0 means the closer the pairwise community similarity is.

## Additional Information and Declarations

### Data Availability

The following information was supplied regarding data availability: Sequence Read Archive (SRA) database of NCBI, accession number SRP048917.
