# Supplementary figures and images for: Microbial communities in the reef water at Kham Island, lower Gulf of Thailand
Source: PeerJ. 2017 Aug 14;5:e3625. doi: 10.7717/peerj.3625 (PMC5560237; doi:10.7717/peerj.3625)

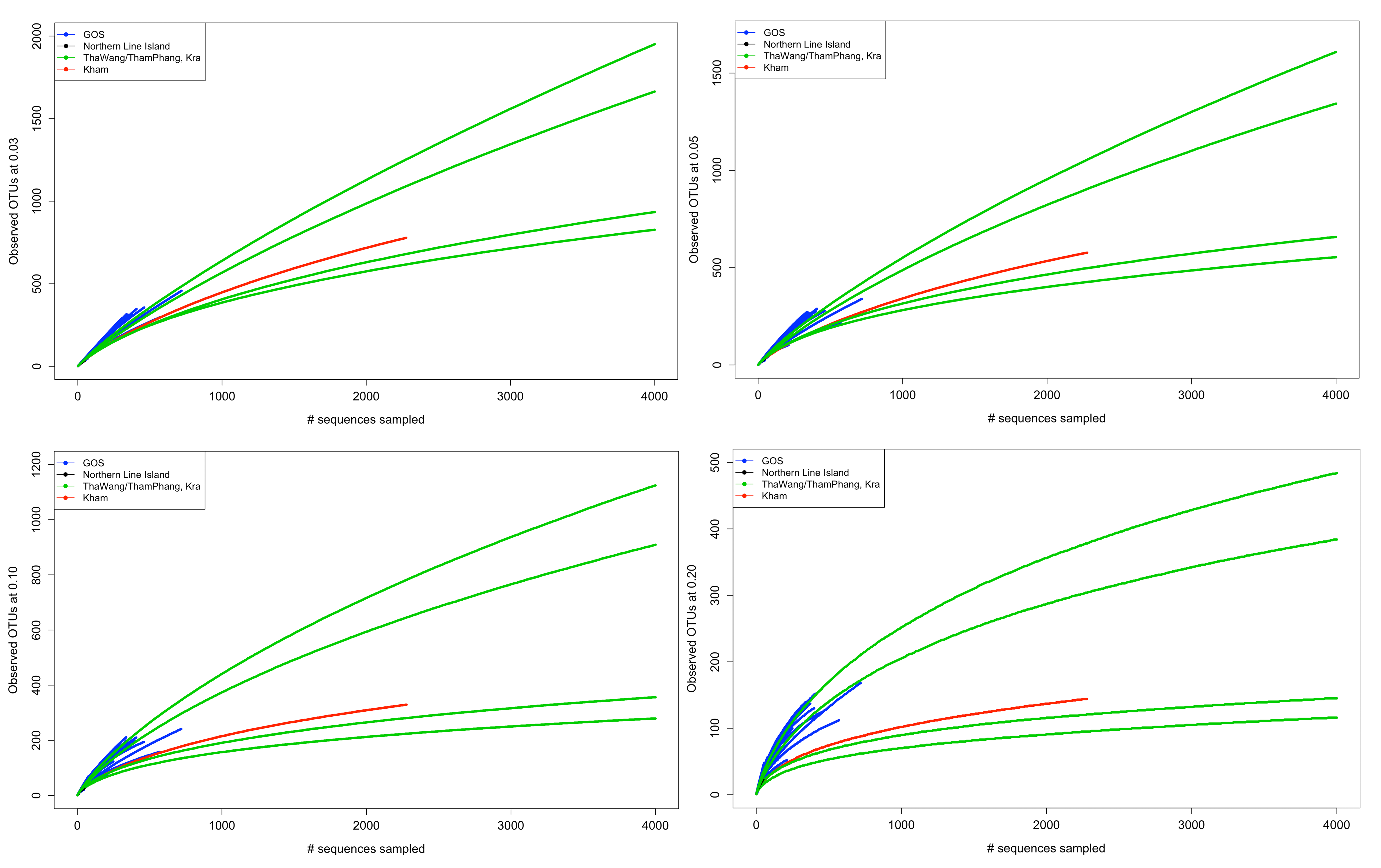

Supplement: Figure S1 — Different color lines represent the number of annotated 16S rDNA sequencing reads in other published data (Tha Wang, Tham Phang, Kra summer, Kra winter, Northern Line Islands, and random selected GOS). [file peerj-05-3625-s001.png]

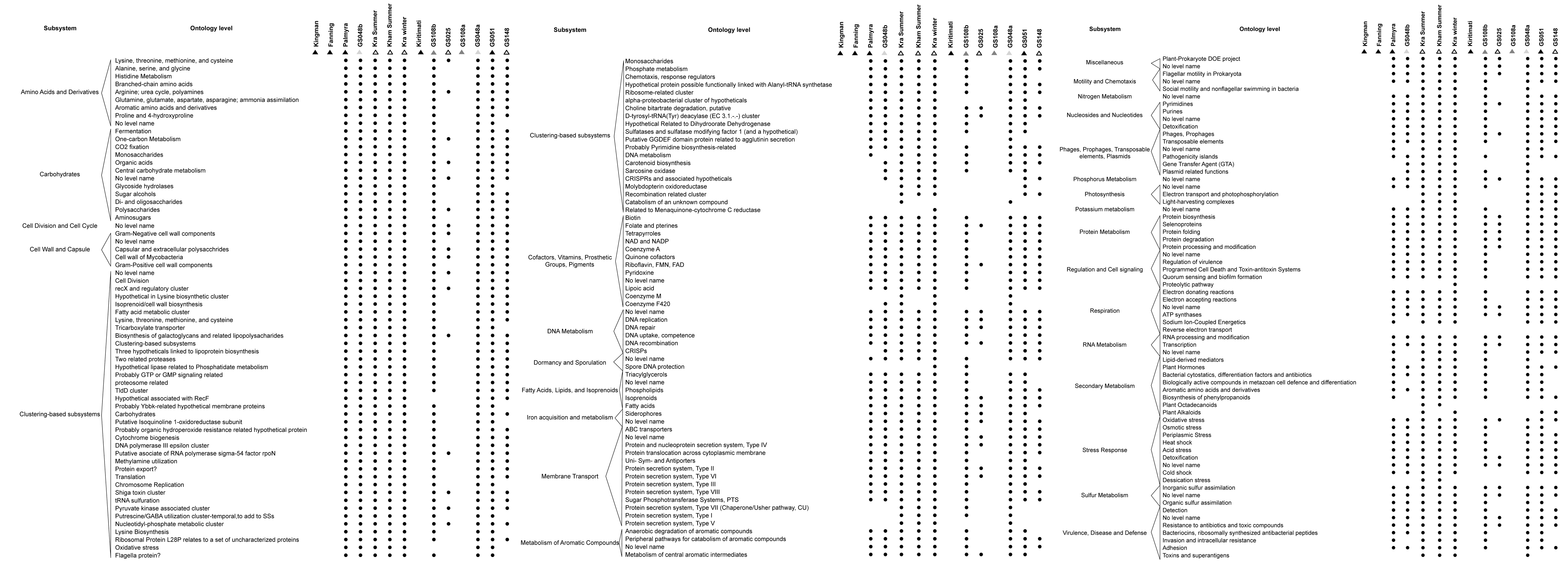

Supplement: Figure S2 — Metabolic potentials of the prokaryotic communities among coral reef sites, categorized by subsystems and functional groups (ontology level). [file peerj-05-3625-s002.png]

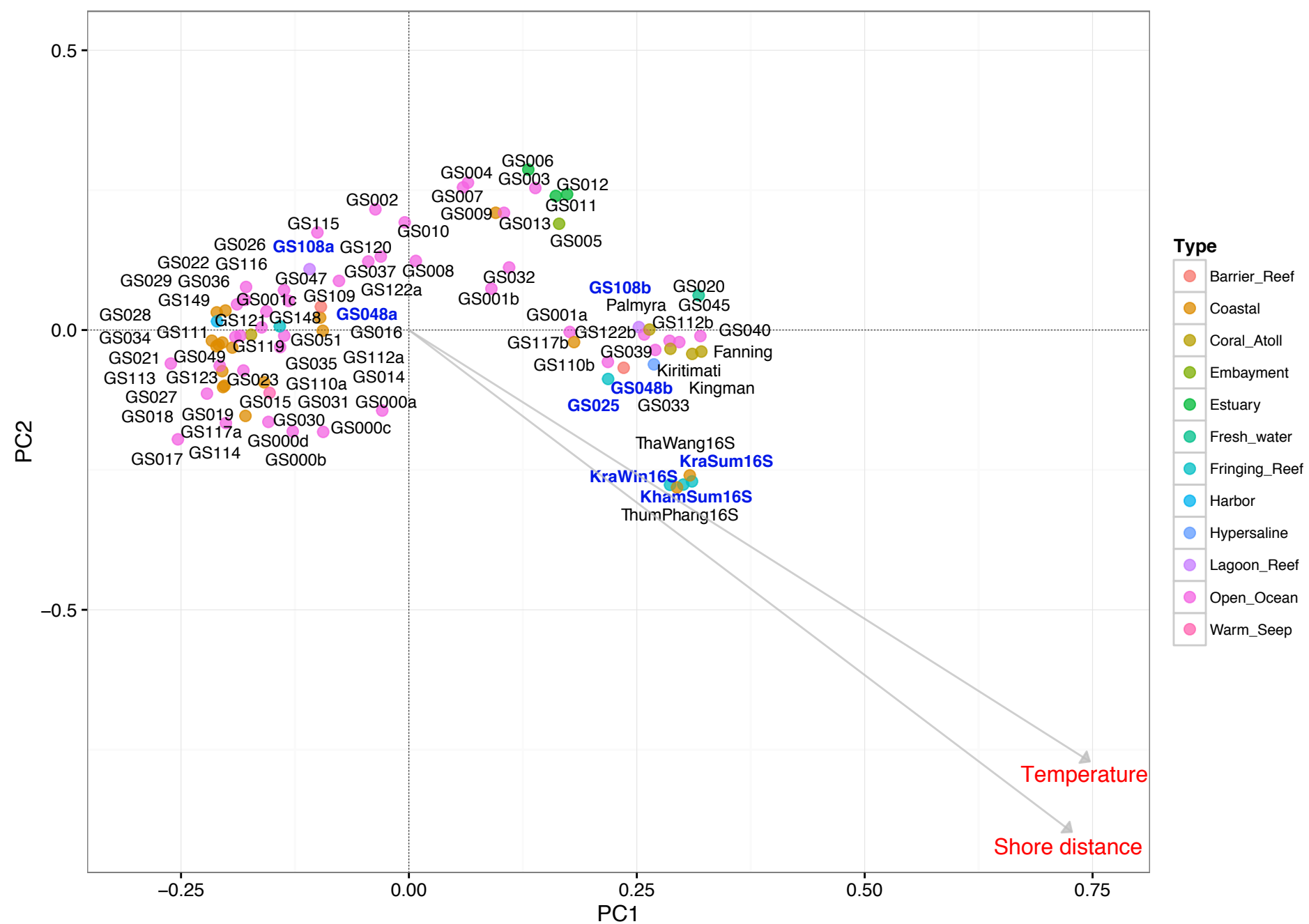

Supplement: Figure S3 — Different color dots denote different types of ocean habitats, e.g., fringing reef, barrier reef, lagoon reef, and coastal. The direction of vector infers the direction of the effect, the length infers the strength of the association in that direction, and the color grey indicates the level of 0.01 < p-value ≤0.05. Vector for salinity has p-value >0.05, and thus was omitted. [file peerj-05-3625-s003.pdf]
