# Supplementary material for: Microbial communities in the reef water at Kham Island, lower Gulf of Thailand
Source: PeerJ. 2017 Aug 14;5:e3625. doi: 10.7717/peerj.3625 (PMC5560237; doi:10.7717/peerj.3625)
Supplement: Table S1 — Italic sequence denotes 8-nt pyrotagged sequence. [file peerj-05-3625-s004.docx]

**S1 Table. Pyrotagged 16S and 18S rRNA genes universal primers. Italic sequence denotes 8-nt pyrotagged sequence.**

| **Sample names** | **Forward primers (5′-3′)** | **Reverse primers (5′-3′)** |
| --- | --- | --- |
| 16S summer | *TAGCACAC*ACTCCTACGGGAGGCAGCAG | *TAGCACAC*TACCAGGGTATCTAATC |
| 18S summer | *AGCATCAC*CTGGTTGATCCTGCCAGT | *AGCATCAC*ACCAGACTTGCCCTCC |
| 18S winter | *AGATAGCG*CTGGTTGATCCTGCCAGT | *AGATAGCG*ACCAGACTTGCCCTCC |
