# Supplementary material for: Microbial communities in the reef water at Kham Island, lower Gulf of Thailand
Source: PeerJ. 2017 Aug 14;5:e3625. doi: 10.7717/peerj.3625 (PMC5560237; doi:10.7717/peerj.3625)
Supplement: Table S2 — The dissimilarity index ranges 0 to 1, in which the closer to 0 means the closer the pairwise community similarity is. [file peerj-05-3625-s005.docx]

**S2 Table. Pairwise dissimilarity matrics of Kham summer (A) 16S rDNA and (B) 18S rDNA profiles, to the top 5 highest similarity matric sites.** The dissimilarity index ranges 0 to 1, in which the closer to 0 means the closer the pairwise community similarity is.

**A.**

| Comparison | | Morisita-Horn | Thetayc |
| --- | --- | --- | --- |
| Kham Summer | Kra Winter | 0.4166 | 0.5881 |
| Kham Summer | Kra Summer | 0.8323 | 0.9085 |
| Kham Summer | GS048b | 0.9789 | 0.9894 |
| Kham Summer | Palmyra | 0.9790 | 0.9894 |
| Kham Summer | GS108b | 0.9850 | 0.9925 |

**B.**

| Comparison | | Morisita-Horn | Thetayc |
| --- | --- | --- | --- |
| Kham Summer | Kham Winter | 0.9956 | 0.9978 |
| Kham Summer | Kra Summer | 0.0215 | 0.0421 |
| Kham Summer | Kra Winter | 0.9997 | 0.9998 |
| Kham Summer | Tha Wang | 0.9200 | 0.9583 |
| Kham Summer | Tham Phang | 0.0179 | 0.0353 |
